# Invisible Pain, Visible Inequalities: Gender, Social Agency, and the Health of Women with Fibromyalgia

**DOI:** 10.3390/healthcare13233143

**Published:** 2025-12-02

**Authors:** Andrea Lizama-Lefno, Ángel Roco-Videla, Erick Atenas-Núñez, Nelia González-Droguett, María Jesús Muñoz-Yánez, Sergio V. Flores-Carrasco

**Affiliations:** 1 Facultad de Ciencias de la Rehabilitación y Calidad de Vida, Universidad San Sebastián, Santiago 8420524, Chile; andrea.lizama@cloud.uautonoma.cl; 2 Dirección de Desarrollo y Postgrados, Universidad Autónoma de Chile, Santiago 7500138, Chile; 3 Facultad de Ingeniería, Universidad Católica de la Santísima Concepción, Concepción 4090541, Chile; aroco@ucsc.cl; 4 Dirección de Investigación y Doctorados, Universidad Gabriela Mistral, Santiago 7500533, Chilemariajesus.munoz@ugm.cl (M.J.M.-Y.); 5 Fundación Núcleo de Investigación DOLMEN, El Director 6000, Of. 207, Las Condes, Santiago 7580023, Chile; 6 Instituto de Estudios Avanzados (IDEA), Facultad de Humanidades, Universidad de Santiago, Santiago 9170022, Chile; 7 Centro de Investigación en Medicina de Altura, Universidad Arturo Prat, Iquique 110939, Chile; 8 Facultad de Ciencias de la Salud, Universidad Católica Silva Henríquez, Santiago 8280354, Chile

**Keywords:** fibromyalgia, social support, health inequalities, sense of agency, social determinants of health, mental health, symptomatology

## Abstract

**Background**: Fibromyalgia (FM) is a chronic nociplastic pain condition predominantly affecting women. Although often addressed within biomedical frameworks, its structural and relational dimensions remain underexplored. This study examined how social, familial, and economic factors are associated with symptom severity and self-perceived mental health among women with FM, with particular emphasis on social participation and perceived discrimination. **Methods**: A cross-sectional study was conducted among women diagnosed with FM in Chile. Participants were recruited through patient organizations and community networks and completed a self-administered questionnaire covering biopsychosocial aspects of FM. Multifactor ANOVA models were used to explore associations between social and familial variables and symptom count and perceived mental health. **Results**: Participants were mostly middle-aged women who reported a high burden of symptoms and moderate levels of self-perceived mental health. Involvement in FM-related organizations was significantly associated with differences in symptom perception and better mental health, while perceived discrimination was linked to poorer mental health outcomes. **Conclusions**: Social participation and supportive environments emerge as potential determinants of health among women with FM. Primary care providers should adopt social and gender-sensitive approaches that acknowledge the influence of discrimination, economic vulnerability, and support networks in shaping the illness experience.

## 1. Introduction

Fibromyalgia (FM) is classified in the ICD-11 under Chronic Widespread Pain (code MG30.01) [1]. It is characterized by widespread musculoskeletal pain, fatigue, sleep and cognitive disturbances as well as central nervous system hypersensitivity [2,3,4]. Its estimated prevalence ranges from 0.2% to 4.7%, predominantly among women [5,6,7,8]. Although diagnosis relies on clinical criteria in the absence of identifiable nociceptive causes, FM is increasingly recognized as a nociplastic pain condition shaped by psychological and social factors [9]. The absence of reliable biomarkers complicates early detection and continues to fuel medical and social debate [10].

Despite patients frequently describing fibromyalgia as an unpredictable and exhausting illness that profoundly affects daily functioning and emotional well-being [7], FM remains underacknowledged due to its invisibility within dominant biomedical paradigms. The lack of objective markers sustains perceptions of FM as an “unreal” or psychosomatic condition [11,12], producing clinical and social delegitimization. Many women experience skepticism from healthcare providers and institutions, resulting in isolation, neglect, and anger [13,14,15]. As a result, patients often need to advocate for themselves to gain recognition and appropriate care, which can further intensify emotional fatigue. These dynamics reflect historical gender biases that pathologize women’s pain and reinforce stigma and discrimination, underscoring the need for a gender-based approach in understanding FM [16,17].

Beyond physical pain, women with FM face substantial psychosocial stressors. The condition demands sustained commitment and self-management, yet many patients feel unprepared for the therapeutic process. Diagnosis and prognosis are often perceived as discouraging [10,18], while symptom unpredictability, persistent pain, and daily functional limitations generate chronic stress, amplified by economic precarity and workplace discrimination [16].

The impact of FM is further intensified by psychological factors such as fear of movement, catastrophizing, low self-efficacy, helplessness, and an external locus of control [19,20]. These vulnerabilities interact with the overload of paid, domestic, and caregiving responsibilities typical of women’s daily lives. This cumulative burden aggravates symptoms, disrupts rest, and limits self-care, generating a cycle of pain, guilt, and negative self-perception in social and family roles [21]. Consequently, women with FM often experience increased anxiety, depression, and pain perception [22,23]. Beyond individual suffering, FM disrupts social and family life, restricting participation in everyday, leisure, and intimate activities [24,25,26]. Because women often hold central caregiving roles, their symptoms may be misinterpreted as neglect, straining family bonds and reducing social support. The chronic and unpredictable course of FM also undermines job stability and generates household financial strain due to economic insecurity and healthcare costs [27,28]. Thus, FM emerges not only as a health condition but also as a social and cultural issue.

In this context, the present study adopts a quantitative epidemiological approach to examine how social and gender-related factors are associated with the experience of FM. By analyzing social, economic, and familial variables in relation to symptom severity and mental health outcomes, it seeks to highlight the consistent influence of socio-structural determinants across the illness trajectory and to contribute to a comprehensive framework that challenges the biomedical paradigm.

Recent research reveals that social factors such as family dynamics, partnership quality, and perceived loneliness not only result from FM but also shape its symptom severity [20]. These findings align with the broader framework of social determinants of health, emphasizing how economic stability, social recognition, and support networks influence illness trajectories [29]. Peer support among women with FM operates as a protective factor, promoting emotional validation, coping, and collective resistance to medical and social delegitimization [30,31].

To move beyond biomedical reductionism, this study adopts a sociological perspective that considers the interaction of physiological, psychological, and social dimensions. A medical sociology approach enables analysis of how structural factors -gender, social participation, discrimination, and economic inequalities- are related to the onset, experience, and treatment of FM. Accordingly, the study examines how social and familial variables are associated with symptom severity and perceived mental health, emphasizing the need for intersectional frameworks that address the social and gender-based determinants of women’s experiences with FM.

The growing body of qualitative and phenomenological research on women’s experiences with FM has revealed the biopsychosocial and structural nature of the condition [16,18,32,33,34]. This work has legitimized a critical perspective recognizing that FM-like other conditions related to chronic pain and women’s mental health-cannot be fully understood without considering gender, power relations, and systemic inequalities as social determinants of health [35]. Integrating qualitative and quantitative approaches strengthens the comprehension of FM by linking lived experience with measurable social patterns, allowing for the testing and generalization of findings.

This quantitative study provides an explanatory analysis that complements previous qualitative findings, offering epidemiological support for women’s lived experiences with FM. By examining social, economic, and familial variables, it demonstrates how socio-structural determinants shape physical and mental health perceptions, reinforcing a comprehensive approach beyond the biomedical paradigm. Guided by this framework, the study hypothesizes that (a) women reporting higher social participation, satisfaction with social life, and family support will show fewer symptoms and better mental health, whereas (b) perceived discrimination and lower economic satisfaction are expected to relate to greater symptom severity and poorer mental-health outcomes.

## 2. Materials and Methods

We conducted the study in Chile, a country where gender disparities and socioeconomic inequalities create a relevant context for examining the social dimensions of fibromyalgia. This setting enabled us to analyze how social participation, discrimination, and family dynamics are associated with women’s health outcomes, while maintaining conceptual relevance to other societies with comparable structural characteristics.

### 2.1. Design

To analyze how social and family factors are associated with the experience of fibromyalgia, we adopted a quantitative and cross-sectional design. Unlike biomedical approaches that focus exclusively on symptomatology and pathophysiological mechanisms, our study prioritized a biopsychosocial perspective, considering how the social context shapes the lived experience of illness. We collected data through a self-administered online questionnaire that explored the biopsychosocial dimensions of fibromyalgia.

We developed the questionnaire to collect clinical, psychological, social, and occupational information relevant to the condition. The instrument was based on a comprehensive review of the scientific literature and on exploratory interviews with patients and health professionals specializing in rheumatology, psychology, and rehabilitation.

We carried out content validation using the Delphi method with a multidisciplinary panel of eight experts from medical and social science fields. In two iterative rounds, the experts independently assessed each item for clarity, relevance, and appropriateness. Items that achieved at least 80% consensus were retained, and we incorporated the qualitative feedback until the panel reached full agreement, as described in our previous work [36].

The final questionnaire includes 60 items grouped into eight thematic domains: (1) general background (diagnosis, age, sex, nationality); (2) sociodemographic background (marital status and family composition); (3) medical history (perceived health, symptoms, disease duration, comorbidities, and treatments); (4) clinical history (type and frequency of medical consultations and treating specialist); (5) mental health and emotions (psychological consultation, therapy experience, and emotional coping); (6) family background (family support, domestic roles, and childhood experiences); (7) social background (ethnic identity, participation, discrimination, and social satisfaction); and (8) occupational background (paid and unpaid work, work–family balance, and disability-related retirement).

The questionnaire includes dichotomous, multiple-choice, and Likert-type items (4- or 5-point scales, depending on the section). We designed it to enable the integrated analysis of medical, psychological, and social dimensions among individuals living with fibromyalgia.

We performed a data consistency analysis on the dataset. The procedure involved checking for missing values, identifying out-of-range responses in Likert-type items, detecting logical inconsistencies between related variables, and summarizing numeric ranges.

We assessed internal consistency for each questionnaire domain using Cronbach’s alpha (α) and McDonald’s omega (ω) coefficients. The analysis included only Likert-type items, excluding single-item domains from reliability estimation. The evaluated domains were Medical Background, Family Background, Social Background, and Work Background.

### 2.2. Participants

We recruited participants through a non-probabilistic sampling strategy, using an open call disseminated via social media by two fibromyalgia-related organizations. Selection was non-random: of the 663 questionnaires received, we included 544 women residing in Chile who met the criteria of having a fibromyalgia diagnosis, being over 18 years old, and providing online informed consent. The focus on women reflected not only the higher prevalence of fibromyalgia in female populations but also our aim to examine how gender shapes the experience of chronic pain and interactions with the healthcare system.

### 2.3. Materials and Procedure

Participants rated their physical health, family support, satisfaction with social life, and work–family balance through single Likert-type items with 4- or 5-point response scales, depending on the questionnaire section (items 9.1, 38, 54, and 59, respectively). Higher scores reflected more favorable evaluations in each domain. The mean age of participants was 44 years (SD = 10.86).

We measured two main dependent variables. The number of perceived symptoms corresponded to the sum of 40 dichotomous items (presence/absence) from the Symptom Severity (SS) Score section of the questionnaire (item 10). Each reported symptom received a value of 1, and the total number of reported symptoms indicated overall symptom burden. Self-perceived mental health was measured through a single Likert-type item (item 9.2) rated on a 5-point scale ranging from 1 (“very poor”) to 5 (“excellent”).

We began the data analysis with a descriptive examination of means, frequencies, and proportions, including participants’ age distribution. Subsequently, we applied six multifactorial ANOVA models to identify significant associations between social and family factors and both mental health and symptomatology outcomes. We examined group differences using one-way ANOVA applied to composite scores derived from Likert-type items.

Although Likert-type items are ordinal, composite scores from 4- to 5-point scales can validly be treated as continuous, as supported by empirical evidence demonstrating the robustness of parametric tests under such conditions [37]. Then, we replicated the models excluding non-significant variables to refine the identification of factors most strongly associated with the illness experience. Finally, we developed a composite model integrating the social and family dimensions that showed significant associations (Table 1). A statistical significance threshold of *p* < 0.05 was applied to hypothesis testing. To improve clarity and avoid redundancy, only the full models (M3 and M6), which integrate both the social and family dimensions, are reported in detail.

To address potential confounding by clinical factors, an additional multiple linear regression model was fitted to examine the number of reported symptoms while controlling for disease duration and pharmacological treatment. The adjusted model included the same social and familial predictors analyzed in the main models (social participation, satisfaction with social life, satisfaction with family economic situation, perceived family support, and perceived discrimination) together with clinical covariates: time since diagnosis (P1.1) and the number of medications currently used (Qfarmacos).

This study was conducted in strict compliance with ethical standards and in accordance with both international and national regulations aimed at protecting the rights and dignity of participants. Specifically, the research adhered to the guidelines outlined in the Declaration of Helsinki [38], ensuring the protection of participants’ autonomy, privacy, and well-being throughout the study. Furthermore, the study complied with Chilean Law 21.096, which governs the protection of personal data, as well as Law 20.584, which regulates the process of obtaining informed consent in healthcare settings, both of which are fundamental in ensuring the ethical management of sensitive information under Chilean legislation [39,40]. Ethical approval for the study was granted by the Institutional Ethics Committee of the University of Santiago de Chile (Report No. 293), which reviewed the study’s design and methods to ensure that participants were fully informed about the research objectives, the risks involved, and the voluntary nature of their participation.

In line with a socially responsible research approach, this study promotes social justice by highlighting the voices of vulnerable groups, specifically focusing on the experiences of women suffering from a chronic, complex, and feminized condition that is often accompanied by medical violence and social discrimination. The study was committed to ethical data collection, ensuring that participants were treated with respect and sensitivity to their needs, despite the virtual nature of the questionnaire.

## 3. Results

The dataset exhibited overall good structural consistency. Missing data primarily corresponded to conditional or context-dependent items. No out-of-range Likert responses were identified, and logical inconsistencies were rare. Overall, the database was deemed suitable for subsequent statistical analyses, pending the review of variables with substantial missingness.

The Medical Background domain demonstrated good internal consistency (α = 0.74; ω = 0.75). The Family Background domain showed moderate reliability (α = 0.59; ω = 0.62), indicating some heterogeneity among items. The Social Background domain achieved acceptable reliability for a two-item construct (α = 0.70; ω = 0.70), whereas the Work Background domain, consisting of a single item, was not eligible for reliability estimation. Descriptive analyses of individual items P31 and P34 yielded mean scores of 1.31 (SD = 0.58) and 2.52 (SD = 1.42), respectively, reflecting distinct response patterns (Table 2). Overall, the reliability coefficients support the adequacy of the instrument for exploratory and descriptive research on the biopsychosocial characterization of individuals with fibromyalgia.

### 3.1. Descriptive Characteristics of the Sample

Regarding their perceived health status, participants reported an average of 14.55 symptoms in the past week (SD = 5.87), with a range from 0 to 23 symptoms. Additionally, their self-assessed mental health had a mean score of 2.13 (SD = 0.76) on a scale from 1 to 5. The sociodemographic profile of the participants is summarized in Table 2. Briefly, the sample consisted of adult women residing in different regions of Chile, most of whom identified as Chilean.

Descriptive statistics for the dependent and independent variables included in the regression and ANOVA models are presented in Table 3. The table summarizes the central tendency and variability of each variable across the social and family domains, including indicators of symptom burden, perceived mental health, social participation, recognition, discrimination, and family support.

From a social perspective, satisfaction with one’s social life had a mean score of 2.8, while perceived social recognition averaged 2.9, both measured on a 1-to-5 scale. These values suggest that participants are moderately satisfied with both their social life and the social recognition they perceive. In terms of participation in support networks, 15.4% of participants were involved in a fibromyalgia-related social organization. Furthermore, 27% of women reported experiencing discriminatory treatment on a regular basis.

Within the family domain, 54% of women indicated that their family was highly or largely aware of their illness, whereas 46% reported little or no awareness. Regarding household economic stability, 67% expressed satisfaction (very satisfied, satisfied, or moderately satisfied), compared to 33% who reported some level of dissatisfaction (dissatisfied or very dissatisfied). In terms of family support, 72% felt supported to some degree, while 28% reported feeling little or no support. Finally, 83% of participants expressed satisfaction with their current romantic relationship, while 17.2% reported feeling little or no satisfaction.

### 3.2. Predictors of Symptom Count

**Figure 1:**
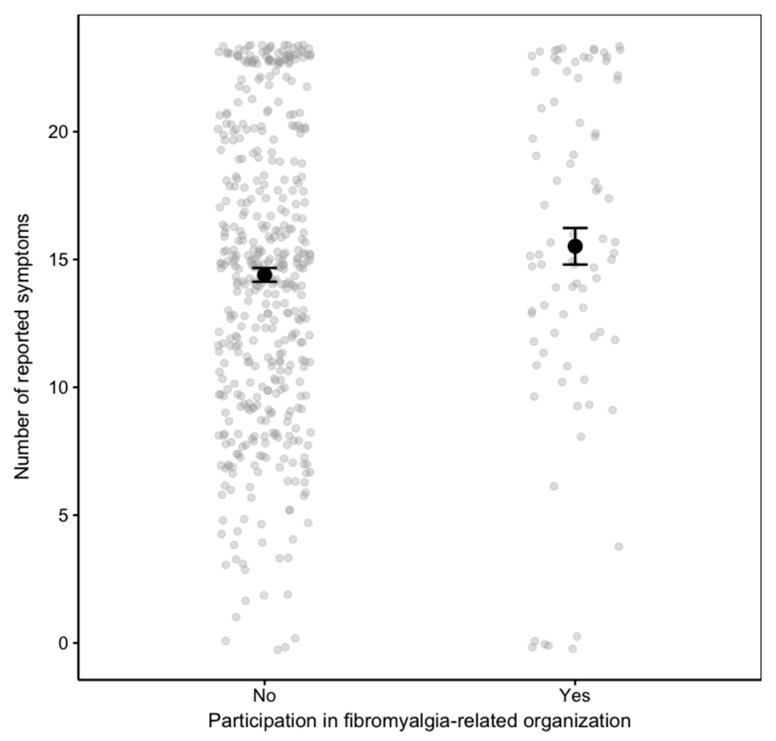
Mean number of self-reported fibromyalgia symptoms according to participation in fibromyalgia-related organizations (P51).

In the analysis of predictors for the number of symptoms experienced during the previous week, involvement in a fibromyalgia-related social organization (P51) emerged as the only significant variable in the integrated model combining social and familial dimensions (Table 4). This finding highlights that affiliation with a disease-specific social network is significantly associated with differences in symptom perception, reflecting the potential influence of social context on the illness experience. Although statistically significant in the multivariate model, the magnitude of this association was modest. Participants engaged in fibromyalgia-related organizations reported a slightly higher mean number of symptoms (M = 15.5, SD = 6.5) compared to non-participants (M = 14.4, SD = 5.7), corresponding to a small standardized mean difference (Cohen’s d = −0.19, 95% CI [−0.42, 0.04]). A Mann–Whitney U test confirmed a marginally significant difference between groups (W = 16,190, *p* = 0.047). As shown in Figure 1, the distributions of symptom counts largely overlap, suggesting that the observed difference, while statistically significant, has limited practical relevance.

### 3.3. Predictors of Self-Perceived Mental Health

When considering only the social dimension (Table 5), self-perceived mental health was mainly influenced by satisfaction with social life (P54), which emerged as the most consistent social correlate of mental well-being. This variable showed a significant effect both as an independent predictor (*p* = 0.005) and in interaction with social participation (P51) (*p* = 0.006), underscoring the relevance of active engagement in social networks and community-based support. The perception of discriminatory treatment (P53) was also independently associated with poorer self-perceived mental health (*p* = 0.008), reflecting the detrimental impact of social stigma on psychological well-being.

**Figure 2:**
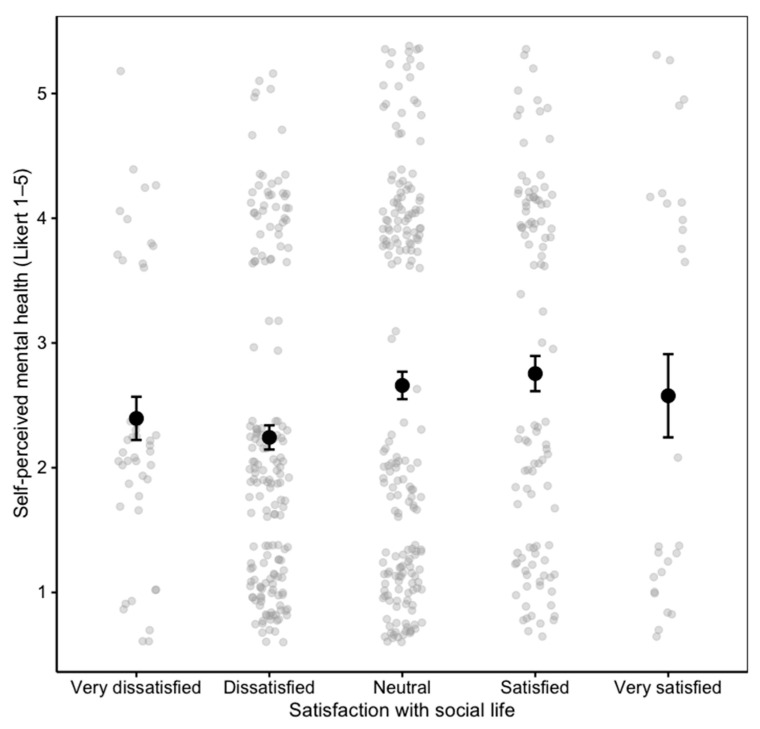
Self-perceived mental health according to satisfaction with social life.

In the integrated ANOVA model that combined social and family dimensions, satisfaction with social life (P54) remained a significant predictor of self-perceived mental health (*p* = 0.001), together with its interaction with satisfaction with the family’s economic situation (P37) (*p* = 0.045). This interaction indicates that the positive influence of satisfaction with social life on mental health becomes stronger among participants who were more satisfied with their family’s economic situation. In other words, greater social satisfaction is associated with better self-perceived mental health, and this association is amplified when perceived financial stability is higher. Descriptive and nonparametric analyses confirmed that higher satisfaction with social life was associated with better self-perceived mental health, although the effect size was small (Cohen’s *d* = −0.19, 95% CI [−0.42, 0.04]) and the Kruskal–Wallis test did not reach statistical significance (*p* = 0.075). These results suggest that, while statistically robust in the parametric model, the practical magnitude of the association is modest, highlighting the complex and multifactorial nature of mental health in women with fibromyalgia (Figure 2).

After controlling for clinical covariates, the adjusted multiple linear model explained 9.4% of the variance in the number of reported symptoms (F(7, 524) = 7.73, *p* < 0.001). Satisfaction with social life (P54; β = 0.67, *p* = 0.016) and perceived family support (P38; β = 0.54, *p* = 0.016) remained statistically significant predictors, while the number of medications currently used (Qfarmacos; β = 0.61, *p* < 0.001) showed a strong positive association with symptom count. Other variables, including social participation (P51), family economic satisfaction (P37), perceived discrimination (P53), and disease duration (P1.1), were not significant in the adjusted model. These findings confirm that the association between social factors and symptom perception persists independently of clinical burden.

## 4. Discussion

This research highlights the close relationship between social integration, support networks and agency, and the perceived health status and well-being of women with fibromyalgia. Women who actively participate in support networks and disease-related organizations tend to report better physical and mental health, a pattern that may reflect greater autonomy, self-care capacity, and perceived influence over their care. These findings illustrate how social and cultural structures shape the ways in which women cope with chronic illnesses, emphasizing the fundamental role of collectivity and empowerment in their well-being [33]. The literature on gender and health has extensively documented that support networks provide emotional support and enable women to challenge the medicalization of their experiences, gaining greater control over their bodies and health-related decisions [41,42].

The use of a non-probabilistic, voluntary sampling strategy may introduce self-selection bias and limit generalizability. However, this approach is common and often unavoidable in studies of chronic or hard-to-reach populations such as women with fibromyalgia, where probabilistic sampling is logistically unfeasible. Recruitment through fibromyalgia-related organizations may also have favored the inclusion of more socially active participants. Consequently, the association between organizational participation and better self-perceived health should be interpreted cautiously, as it likely reflects the supportive role of these networks rather than a causal relationship. Moreover, as this was a cross-sectional study, the observed associations cannot be interpreted as causal. The relationships identified reflect correlations between social and family factors and health outcomes rather than directional effects. The term predictor is used solely in its statistical sense within the ANOVA framework and does not imply causality.

The relatively low R-squared values observed in some models indicate that the social and family dimensions analyzed account for only part of the variance in symptom perception and self-perceived mental health. This result aligns with the multifactorial nature of fibromyalgia, in which biological, psychological, and contextual variables interact in complex ways. Factors such as pain intensity, fatigue, sleep quality, and coping strategies -though relevant-were not included in the present dataset and may contribute to unexplained variance.

After adjustment for disease duration and medication use, satisfaction with social life and perceived family support remained significant predictors of symptom count, indicating that social context influences symptom perception beyond clinical burden. The positive association between number of medications and symptom count likely reflects higher clinical severity and treatment complexity. These results confirm the robustness of the social dimension, underscoring the relevance of relational and contextual factors in the experience of fibromyalgia [43].

The role of participation in fibromyalgia-related organizations is a key variable statistically associated with health status, expressed in the number of symptoms and perceived mental health. The literature has emphasized that peer groups constitute essential spaces for illness validation, collective identity construction, and access to psychosocial coping strategies [42,44]. This finding is significant as it reinforces that the perception and experience of symptoms are not merely physiological phenomena but are also shaped by access to support networks, recognition, and social agency-that is, the capacity to act collectively to transform reality and influence their social and political environment [21].

Collective agency, social support and recognition, the exchange of informational, therapeutic, and socio-emotional resources, and empowerment emerge as key dimensions in the lived experience and perceived well-being of women with fibromyalgia. In a context where chronic pain and fatigue are often delegitimized both medically and socially, the possibility of sharing experiences with other women in similar situations translates into greater autonomy and a redefinition of the illness [45].

Another central aspect identified in this study is the relationship between the perception of discrimination and mental health. Women who have experienced discriminatory treatment report poorer self-perceived mental health. The literature has documented that women with fibromyalgia face high levels of stigmatization due to the invisibility of their symptoms, the lack of objective biomarkers, and gender biases in healthcare [46,47,48]. These biases manifest in a tendency to minimize women’s pain or attribute it to psychological factors, which not only affects the care they receive but also their own perception of the legitimacy of their illness [49]. Feeling validated and recognized, not only in the medical field but also socially, is an essential component of emotional and mental well-being.

This finding highlights how discrimination and structural gender violence operate as key social factors associated with health outcomes, creating a double vulnerability for women with fibromyalgia. First, the medical and social stigmatization they face not only strips them of the legitimacy of their own pain experience but also limits their access to timely diagnosis and appropriate treatments. The literature indicates that women with chronic illnesses of unclear origin are more likely to receive misdiagnoses, inadequate treatments, or be outright dismissed in medical consultations under the suspicion that their distress is exaggerated or psychosocially driven. This phenomenon is a manifestation of gender bias in medicine, which has historically interpreted the female body as more susceptible to hysteria, emotionality, or somatization, perpetuating forms of medical violence that is closely linked to women’s mental and physical health [50]. This dynamic has been referred to by some scholars as “clinical epistemic injustice” [51].

This pattern of clinical bias is not unique to fibromyalgia but extends to other pain-related conditions that predominantly affect women, such as endometriosis and chronic pelvic pain syndromes. These examples further illustrate the systematic undervaluation of women’s pain within biomedical practice and underscore the need to take women’s pain more seriously across all domains of healthcare. Self-perceived mental health deteriorates not only due to the burden of the illness itself but also due to the chronic stress stemming from the constant need to prove the legitimacy of their condition, whether to healthcare professionals, in the workplace, or even within their own families [52,53].

Moreover, the economic-family variable also emerges as a predictor of health, particularly mental health. Economic insecurity is associated with higher levels of stress and anxiety, whereas a better financial situation improves access to healthcare resources, treatments, and support networks, thereby mitigating the impact of the illness on mental health [54]. In this regard, the study’s results show that the effect of perceived family economic status on mental health is significant, particularly when it interacts with social life satisfaction, that is, with their perception of their social relationships and interactions. Mental health deteriorates when family economic conditions are adverse; in this context, the perception of recognition, emotional support, and social well-being helps mitigate this deterioration, whereas, conversely, the perception of invisibility, neglect, and social exclusion exacerbates mental health problems. Thus, in the face of economic adversity, it becomes essential to activate support networks and foster social integration and well-being.

The results related to the economic-family variable are particularly relevant in the context of a health condition that affects work capacity and generates a considerable economic burden on households, increasing the likelihood of family economic deterioration and, consequently, a worsening of the patient’s mental health, further aggravating the already adverse health condition associated with the illness and likely affecting her and her family’s quality of life [55]. In Chile, more than a third of households are headed by a woman (36.8%), and this, together with the fact that women’s working conditions are more precarious in terms of salary and social protection, alongside a progressive increase in single motherhood, implies greater household vulnerability [56]. This reflects what the literature has categorized as the feminization of poverty. This reality is observed across much of Latin America [57].

Ultimately, the health of women with fibromyalgia cannot be understood outside its social, economic, and gendered context. The illness is not merely an individual biomedical experience but is deeply rooted in structures of inequality that intersect multiple axes of oppression. From an intersectional perspective, it is evident that these women’s suffering is not only delegitimized by gender bias in medicine but also by socio-economic conditions that exacerbate their vulnerability, such as job insecurity, the burden of unpaid domestic work, and the lack of equitable access to healthcare. These inequalities do not operate in isolation but intertwine, generating a scenario of dual exclusion: on the one hand, the denial of their pain in the medical sphere and, on the other, the economic and social neglect that perpetuates their marginalization [35]. The absence of an effective social and political response to address these needs is not neutral but rather reflects a system that has historically invisibilized women’s suffering, denying them the support and resources necessary for their relief and the restoration of their quality of life. This structural omission constitutes a form of institutional violence and an impunity-laden violation of their human rights.

From a medical epistemology perspective, the results of this study challenge the hegemonic biomedical perspective on fibromyalgia and contribute to the scientific corpus revealing that, far from being a purely biological condition, fibromyalgia is clearly mediated by social and gender structures [43,45,49]. Recognizing gender inequalities as determinants of health in women with fibromyalgia reinforces the need for comprehensive, context-sensitive, and gender-focused clinical and therapeutic approaches. From a clinical perspective, these findings are relevant for promoting gender equity and improving women’s health and quality of life at both individual and community levels.

Thanks to the contributions of the social sciences, the study of health has undergone a paradigmatic shift in recent decades, moving away from a purely biomedical vision toward a critical and interdisciplinary approach that recognizes the centrality of sociohistorical factors, structural inequalities, power relations, cultural knowledge, and social narratives in the production of health and illness. From this standpoint, sociology, medical anthropology, and critical social psychology have been instrumental in contextualizing the study of FM and highlighting the urgent need for a structural reading that articulates the biological with the social, the individual with the collective, and the medical with the political.

Future studies should employ longitudinal designs to clarify causal pathways between social participation, discrimination, and health outcomes in fibromyalgia. Randomized controlled trials could evaluate the effects of community-based and psychosocial interventions on symptom perception and mental well-being. Cross-cultural and mixed-method research integrating qualitative perspectives is also recommended to capture contextual and experiential dimensions underlying these associations.

## 5. Conclusions

Social participation and support networks appear to be associated with better perceived mental health and lower symptom reports among women with fibromyalgia, highlighting the importance of collective and social dimensions in their well-being. Likewise, experiences of discrimination are associated with poorer self-perceived mental health and well-being, underscoring the need to address social stigma and gender bias in healthcare and community contexts. These findings suggest that strengthening supportive environments and promoting social recognition may contribute to improving quality of life for women living with fibromyalgia, although longitudinal research is required to clarify causal mechanisms.

## Figures and Tables

**Table 1:** ANOVA Models.

Dependent Variable	Independent Variables	
Number of Symptoms	Social Dimension (M1)	(P51) Participation in a fibromyalgia-related organization
		(P52) Perception of social recognition
		(P53) Perception of discriminatory treatment
		(P54) Satisfaction with social life
	Family Dimension (M2)	(P36) Family awareness of the disease
		(P37) Satisfaction with family economic situation
		(P38) Perception of family support
		(P39) Satisfaction with partnership status
	Social Dimension and Family Dimension (M3)	(P51) Participation in a fibromyalgia-related organization
		(P52) Perception of recognition
		(P53) Perception of discriminatory treatment
		(P54) Satisfaction with social life
		(P36) Family awareness of the disease
		(P37) Satisfaction with family economic situation
		(P38) Perception of family support
		(P39) Satisfaction with partnership status
Self-Perception of Mental Health	Social Dimension (M4)	(P51) Participation in a fibromyalgia-related organization
		(P52) Perception of social recognition
		(P53) Perception of discriminatory treatment
		(P54) Satisfaction with social life
	Family Dimension (M5)	(P36) Family awareness of the disease
		(P37) Satisfaction with family economic situation
		(P38) Perception of family support
		(P39) Satisfaction with partnership status
	Social Dimension and Family Dimension (M6)	(P51) Participation in a fibromyalgia-related organization
		(P52) Perception of recognition
		(P53) Perception of discriminatory treatment
		(P54) Satisfaction with social life
		(P36) Family awareness of the disease
		(P37) Satisfaction with family economic situation
		(P38) Perception of family support
		(P39) Satisfaction with partnership status

**Table 2:** Sociodemographic characteristics of participants.

Variable	Category	n	%
Age (years)	Mean (SD) = 43.8 (10.9)	539	-
Region of residence	Metropolitan Region	400	74.2
	Other regions of Chile	139	25.8
Nationality (P6)	Chilean	534	99.1
	Other	5	0.9
Marital status (P7)	Single (no partner)	59	10.9
	Single (with partner)	115	21.3
	Married (no partner)	40	7.4
	Married (with partner)	221	41.0
	Divorced (no partner)	49	9.1
	Divorced (with partner)	44	8.2
	Widowed (no partner)	9	1.7
	Widowed (with partner)	2	0.4
Has children	Yes	407	78.6
	No	107	20.0
Children under 18 years	Yes	237	44.0
	No	287	53.2
Socioeconomic Group Scale (GSE) *	Level 1 (lowest)	108	21.0
	Level 2	157	30.0
	Level 3	133	25.0
	Level 4	99	18.0
	Level 5 (highest)	8	1.5
Time since diagnosis (P1.1)	<1 year	78	14.5
	1–3 years	179	33.2
	3–5 years	111	20.6
	5–10 years	92	17.1
	>10 years	77	14.3

* GSE = Chilean socioeconomic stratification scale (1 = lowest, 5 = highest).

**Table 3:** Descriptive statistics of the dependent and independent variables included in the regression and ANOVA models.

Domain	Code	Question	Scale Type	Range	n	Mean	SD
Dependent Variable	QSintomas	Number of reported fibromyalgia symptoms	Count	0–40	539	14.6	5.88
Dependent Variable	P34	How would you rate your current mental health?	5-point Likert	1–5	539	2.52	1.42
Social Dimension	P51	Do you participate in a fibromyalgia-related group or organization?	Dichotomous	0–1	535	0.155	0.362
Social Dimension	P52	Do you feel socially recognized as a person with fibromyalgia?	5-point Likert	1–5	536	2.93	1.18
Social Dimension	P53	Have you felt discriminated against because of fibromyalgia?	5-point Likert	1–5	538	1.73	0.45
Social Dimension	P54	Overall, how satisfied are you with your social life?	5-point Likert	1–5	539	2.81	1.00
Family Dimension	P36	Is your family aware of your diagnosis?	Nominal (1–3)	1–3	536	2.27	0.88
Family Dimension	P37	How satisfied are you with your family’s economic situation?	5-point Likert	1–5	537	3.14	0.98
Family Dimension	P38	How supported do you feel by your family?	5-point Likert	1–5	538	2.73	1.19
Family Dimension	P39	How satisfied are you with your partnership or marital status?	5-point Likert	1–5	529	2.37	1.18

**Table 4:** Predictors of Symptom Count. Integrative ANOVA Model of Social and Family Dimensions (M3).

Source	Sum of Squares Type III	df	Mean Square	F	*p*	Partial η^2^
Corrected Model	5856.049 a	136	43.059	1.366	0.011 *	0.317
Intercept	25,476.134	1	25,476.134	808.475	0.001 *	0.668
P51	293.293	1	293.293	9.308	0.002 *	0.023
Error	12,636.042	401	31.511			
Total	132,653.000	538				
Corrected Total	18,492.091	537				

a. R-squared = 0.317 (Adjusted R-squared = 0.085). * Statistical significance with a *p*-value < 0.05.

**Table 5:** Predictors of Self-Perceived Mental Health. Integrative ANOVA Model of Social and Family Dimensions (M6).

Source	Sum of Squares Type III	df	Mean Square	F	*p*	Partial η^2^
Corrected Model	190.650 a	282	0.676	1.515	0.001 *	0.633
Intercept	402.626	1	402.626	902.476	0.001 *	0.784
P54	8.138	4	2.034	4.560	0.001 *	0.069
P37 × P54	11.624	15	0.775	1.737	0.045 *	0.095
Error	110.642	248	0.446			NA
Total	2706.000	531				NA
Corrected Total	301.292	530				NA

a. R-squared = 0.633 (Adjusted R-squared = 0.215). * Statistical significance with a *p*-value < 0.05.

## Data Availability

The data supporting the findings of this study are not publicly available due to privacy and ethical restrictions.

## Abbreviations

The following abbreviations are used in this manuscript:

FM	Fibromyalgia
ICD-11	International Classification of Diseases, 11th Revision
ANOVA	Analysis of Variance
SD	Standard Deviation
WHO	World Health Organization
