# Correction: Lizama-Lefno et al. Invisible Pain, Visible Inequalities: Gender, Social Agency, and the Health of Women with Fibromyalgia. *Healthcare* 2025, *13*, 3143

**DOI:** 10.3390/healthcare14060754

**Published:** 2026-03-18

**Authors:** Andrea Lizama-Lefno, Ángel Roco-Videla, Erick Atenas-Núñez, Nelia González-Droguett, María Jesús Muñoz-Yánez, Sergio V. Flores-Carrasco

**Affiliations:** 1 Facultad de Ciencias de la Rehabilitación y Calidad de Vida, Universidad San Sebastián, Santiago 8420524, Chile; andrea.lizama@cloud.uautonoma.cl; 2 Dirección de Desarrollo y Postgrados, Universidad Autónoma de Chile, Santiago 7500138, Chile; 3 Facultad de Ingeniería, Universidad Católica de la Santísima Concepción, Concepción 4090541, Chile; aroco@ucsc.cl; 4 Dirección de Investigación y Doctorados, Universidad Gabriela Mistral, Santiago 7500533, Chilemariajesus.munoz@ugm.cl (M.J.M.-Y.); 5 Fundación Núcleo de Investigación DOLMEN, El Director 6000, Of. 207, Las Condes, Santiago 7580023, Chile; 6 Instituto de Estudios Avanzados (IDEA), Facultad de Humanidades, Universidad de Santiago, Santiago 9170022, Chile; 7 Centro de Investigación en Medicina de Altura, Universidad Arturo Prat, Iquique 110939, Chile

In the published publication [1], there was an error regarding the affiliations for Sergio V. Flores-Carrasco. Sergio V. Flores-Carrasco is not affiliated with the original affiliation 8 “Facultad de Ciencias de la Salud, Universidad Católica Silva Henríquez, Santiago 8280354, Chile”. This affiliation should be removed from the list. The authors state that the scientific conclusions are unaffected. This correction was approved by the Academic Editor. The original publication has also been updated.
